# Controllable pH Manipulations in Micro/Nanofluidic Device Using Nanoscale Electrokinetics

**DOI:** 10.3390/mi11040400

**Published:** 2020-04-10

**Authors:** Jae Suk Park, Jeewhan Oh, Sung Jae Kim

**Affiliations:** 1 Department of Electrical and Computer Engineering, Seoul National University, Seoul 08826, Korea; radon89@snu.ac.kr (J.S.P.); jeewhanoh@snu.ac.kr (J.O.); 2 Inter-university Semiconductor Research Center, Seoul National University, Seoul 08826, Korea; 3 Nano Systems Institute, Seoul National University, Seoul 08826, Korea

**Keywords:** ion concentration polarization, electrokinetics, reversibility, perm-selective ion transportation

## Abstract

Recently introduced nanoscale electrokinetic phenomenon called ion concentration polarization (ICP) has been suffered from serious pH changes to the sample fluid. A number of studies have focused on the origin of pH changes and strategies for regulating it. Instead of avoiding pH changes, in this work, we tried to demonstrate new ways to utilize this inevitable pH change. First, one can obtain a well-defined pH gradient in proton-received microchannel by applying a fixed electric current through a proton exchange membrane. Furthermore, one can tune the pH gradient on demand by adjusting the proton mass transportation (i.e., adjusting electric current). Secondly, we demonstrated that the occurrence of ICP can be examined by sensing a surrounding pH of electrolyte solution. When pH > threshold pH, patterned pH-responsive hydrogel inside a straight microchannel acted as a nanojunction to block the microchannel, while it did as a microjunction when pH < threshold pH. In case of forming a nanojunction, electrical current significantly dropped compared to the case of a microjunction. The strategies that presented in this work would be a basis for useful engineering applications such as a localized pH stimulation to biomolecules using tunable pH gradient generation and portable pH sensor with pH-sensitive hydrogel.

## 1. Introduction

The theory of electrokinetic transportation in microchannels opens a new era for precise diagnostics of bio- and chemical-substances and environmental monitoring, etc. Alternating current (AC) electroosmosis, electrophoresis and dielectrophoresis on microfluidic platform would be representing electrokinetic techniques of such splendid advancements. More recently, sub-micrometer channels enable to single molecular analysis and even an accurate control of ion and biomolecules. Together with the nanofluidic channels and electricity, new nanoscale electrokinetics phenomena have been actively studied for high efficient (bio-)molecular preconcentrator, portable desalination devices, energy harvesting and fluidic circuit elements. The representing phenomenon is ion concentration polarization (ICP). ICP conventionally referred the polarization of electrolyte concentration at the sides of nanoporous membrane under direct current (dc) bias [1,2]. Since the nanoporous membrane has a perm-selectivity, only counter-ions can pass through the membrane, while co-ions are rejected from the membrane [3]. As a result, an electrolyte concentration at the anodic side of membrane largely decreased (i.e., formed an ion depletion zone) and one at the cathodic side increased (i.e., formed an ion enrichment zone) in the case of cation-selective membrane and vice versa [2,4]. Note that ICP is a branch of concentration polarization (CP) phenomenon because they share similar physical origins of perm-selective ion transportation through nanoporous membrane. However, ICP is different from CP with respect to the fluidic regime. CP researchers usually focused on electrokinetic instability, while ICP researches had been studied in nearly zero-Reynolds number environment. This is because CP system usually has length scale greater than 100 μm and ICP system has one below 10 μm. In such circumstances, even the driving mechanism of overlimiting current is different: electrokinetic instability in CP system [5,6,7,8,9,10,11], surface conduction and electroosmotic flow in ICP system [12,13,14,15,16,17,18,19,20]. Since the ion depletion zone in ICP acts as an electrical filter together with zero-Reynolds number characteristics, it has been actively utilized for low abundance biomolecular separation / accumulation device [21,22,23]. If one can extract the solution from the ion depletion zone, one can use it as portable scale electro-desalination / purification [1,24,25,26,27] device. However, shared fundamental aspects of ICP and CP have been actively studied such as vortex generation [9,10,11,28], stabilization [17,29,30,31], mechanism of overlimiting current [12,13,14,16,18,20], phenomenon near nanopores [32,33,34,35] and concentration profile including pH variation [36,37,38], etc.

The ability to manipulate pH change provides great opportunities to integrate ICP applications with bio/chemical reaction or analysis requiring rapid pH change. For example, precise pH control is highly demanded in ICP desalination device because it produces drinking water [1,26,39]. Other crucial examples are biological applications. While proteins in biological samples are sensitive to changes in the temperature, pH and ionic strength, etc., researchers particularly focused on the regulation of pH in order to minimize pH changes due to its critical impact on biological activity. Previous literatures reported that the pH change could be mitigated by reducing the external electric field or increasing salt concentration in the buffer solution [40] and the surface area of electrodes [36]. To minimize pH changes in ICP device, other had demonstrated that continuous extraction of the pH changed fluid before biomolecules were blocked from passing through the ICP barrier [41].

**Figure 1:**
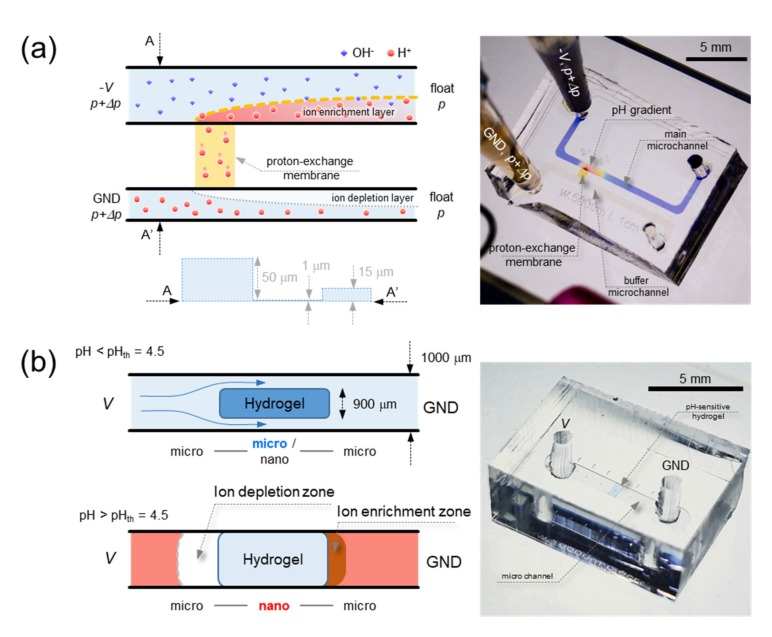
Schematic diagram and fabricated device of (**a**) pH gradient generation by proton-selective transportation through nanoporous membrane and (**b**) pH stimulate effect on the electric current using pH-responsive hydrogel.

However, in this work, we studied how to utilize the inevitable pH changes for useful applications instead of avoiding the pH changes. Adjusting current means controlling the amount of proton transport through proton-exchange membrane, thus, pH value at the proton-receiving side of the membrane can be precisely manipulated (Figure 1a). Note that the proton-receiving side is also known as the ion enrichment zone where electrical conductivity increases so that the analysis about complex electroconvection [42,43,44] is unnecessary in this work. Combined with laminar flow and diffusion characteristics, we could demonstrate the generation of tunable pH gradient as well. Another useful scheme is a sensor using the impact of pH on the electric current with pH-responsive hydrogel. It can significantly change its volume in the response of small alterations of surrounding pH. When the hydrogel swelled inside a microchannel to block it, the microchannel became micro-nano-micro channel structure so that ICP occurred and the electric current dropped significantly (Figure 1b). The results shown in this work were able to demonstrate a phenomenological application. Thus, this concept could be a potential basis for further investigation of the pH utilization. If further developed, one can apply the tunable pH gradient generation for pH stimulation in local area of biomolecules such as muscle cell and the current changes due to pH for the portable pH sensor using pH-sensitive hydrogel, etc.

## 2. Experimental Methods

### 2.1. Device Fabrication

To investigate the effect of proton-selective mass transportation and input flow rate on pH gradient generation, a micro/nanofluidic device made of polydimethyl-siloxane (PDMS) (Sigma-Aldrich, St. Louis, MO, USA) was fabricated as shown in Figure 1a. The dimensions of the microchannel are as follows; the main-microchannel: 500 μm width × 50 μm depth × 1 mm length and buffer-microchannel: 500 μm width × 15 μm depth × 1 mm length. General PDMS fabrication steps were used to fabricate the continuous tunable pH gradient generator [45]. The Nafion for proton-exchange membrane (Sigma-Aldrich, USA) was patterned on the glass substrate based on surface patterning method [46]. Briefly, the solution of Nafion was transferred using a PDMS piece having straight microchannel (50 μm width × 50 μm depth) to a slide glass. Then, the PDMS piece was removed and the glass was heated at 95 °C for 5 min. Nafion transferred glass and the PDMS piece of microchannel network was irreversibly bonded by plasma bonder (CuteMP, Femto Science, Hwaseong, Korea) to a designated position under microscopic observation.

To investigate the pH stimulation effect, a micro/nanofluidic device was fabricated as shown in Figure 1b. It only had one straight microchannel of 1000 μm width × 50 μm depth × 6 mm length. A pH-responsive hydrogel was patterned on the glass substrate based on surface patterning method. The photopolymerizable solutions (76.5 wt % (2- hydroxyethyl) methacrylate (Sigma-Aldrich, USA) + 3 wt % 2,2-dimethoxy-2-phenylacetophenone (DMPA) (Sigma-Aldrich, USA) + 0.5 wt % ethyleneglycoldimethacrylate (EGDMA)(Sigma-Aldrich, USA) + 20 wt % acrylic acid (AA) (Sigma-Aldrich, USA)) were used as the pH-responsive hydrogel. The solution of hydrogel mixture was transferred using a PDMS piece having a straight microchannel (900 μm width × 40 μm depth) to a slide glass. Then, ultraviolet irradiation for 1 min at the dose of 3 mW/cm^2^ (WUV-L50, Daihan Scientific, Seoul, Korea) to polymerize the hydrogel inside the microchannel and then the top PDMS block was detached from the bottom blank PDMS piece. The hydrogel inside the microchannel was brittle and stiff (dry state), but it had sufficient thickness to be transferred onto the device. The slide glass with transferred hydrogel and the PDMS piece of the microchannel were irreversibly bonded by plasma bonder to a designated position under microscopic observation.

### 2.2. Chemical Preparation

The proton transport experiment was mainly performed with two solutions; (i) a HCl solution (10 mM and pH = 3, Sigma-Aldrich, USA) in the buffer microchannel and (ii) a NaOH solution (10 mM and pH = 11. Sigma-Aldrich, USA) in the main microchannel. For the visualization experiments, universal pH indicator (UI-100, Micro Essential, Brooklyn, NY, USA) was mixed with NaOH solution at 1:9 to visualize the pH gradient at the main microchannel. The solution of NaOH:pH indicator = 1:99 was also prepared for the test of the pH indicator’s concentration effect. Any additive was mixed with HCl solution at the buffer microchannel so that only H^+^ can pass through the proton-exchange membrane. In a such condition, we can inject the designated amount of H^+^ to the main microchannel by regulating electrical current.

For pH stimulation experiment, pH-responsive hydrogel was used. The hydrogel maintained its initial volume under threshold pH (pH_th_) = 4.5 and it significantly swelled over the pH_th_. We used a combination of sodium acetate (NaOAc, Sigma Aldrich, USA) and HCl to keep the conductivity equal. 0.13 mM of NaOAc and 0.12 mM of HCl were used for pH = 3.5 and 1.3 mM of NaOAc and 0.2 mM of HCl were used for pH = 5.5 solution. The hydrogel swelled within 3 min, and we assumed that this fast response was mainly due to the size and the shape of the hydrogel block. For visualization of the flow filed, canola-oil droplets were used. Canola-oil (Wesson, Springfield, MA, USA) is ultra-sonicated for 5 min for forming micro-oil droplets (average diameter = ~2 μm). While bulky canola oil is neutral, it became a charged droplet when it broke up below the few micrometers scale. Note that typical tracer such as polystyrene micro particle has high surface charge so that they are mostly rejected from the entrance into an ion depletion zone.

### 2.3. Measurements

For the preliminary test of universal pH indicator’s concentration, the current-voltage characteristics were measured through the Ag/AgCl electrode. Current-voltage responses were driven by a source measure unit (Keithley 236, Keithley Instruments, Cleveland, OH, USA) and recorded by the customized Labview program. An external voltage increased from 0 V to 25 V at 0.2 V/20 s. Note that it was reported that electrolysis is negligible in current values [47,48], while there must be a water dissociation into H^+^ and OH^−^. Furthermore, pH value is not changed if one uses sufficiently large surface area of electrodes [36].

For proton transport experiments, two parameters were mainly tested; proton transportation and external flow rate. By increasing an external current from 0 nA to 1200 nA at 200 nA/120 s to the device, the proton transportation was finely tuned. For active control of the flow rate, a syringe pump (PHD2000, Harvard Apparatus, Holliston, MA, USA) was connected at the reservoirs of each microchannels to infuse liquid. This pressure field and electrical connection were simultaneously applied using a custom-made pipette tip. A stereo microscope (SZ61, Olympus, Tokyo, Japan) with a charge-coupled device (CCD) camera (AcquCAM Pro/U, JNOTIC, Seoul, Korea) was used to optically observe the color of the universal pH indicator inside the microchannel. The custom-coded MATLAB program was used to estimate the corresponding pH values. To obtain reference pH colors, pH of standard pH buffers were measured by a pH meter (Star A215 pH/conductivity meter, Orion) with a microprobe (9810BN Micro pH Electrode, Orion) and the color optically recorded.

For the pH stimulation experiment, I-t curves were obtained by a source measure unit (Keithley 236, USA) with the customized Labview program. An external voltage of 10 V was applied and the current was measured at every 2 s for 2000 s.

All of experiments were repeated more than 10 times and all of results were almost identical in the pH experiment, while there were small deviations in the current experiments.

### 2.4. Image Analysis

The colors of pH variation in the main microchannel in the proton transport experiment were decoded by the customized MATLAB program into RGB (Red, Green, Blue) values. Then the RGB values were transformed into HSV (hue, saturation, value) color model because background light was inevitably included in RGB codes [49]. Thus, all pH variations in this work were expressed as a HSV color model. The snapshots of pH-responsive hydrogel were captured using an inverted microscope (IX53, Olympus, Japan) and the CellSens (Olympus, Japan).

## 3. Results and Discussions

### 3.1. Effect of pH Indicator’s Concentration

Since the universal pH indicator used in this work also has a number of cationic- and anionic-compounds, one need to verify the effect of pH indicator itself on the H^+^ transportation through proton-selective membrane. The color of pH indicator should represent an actual pH value, but should have the minimal effect on the mass transport.

**Figure 2:**
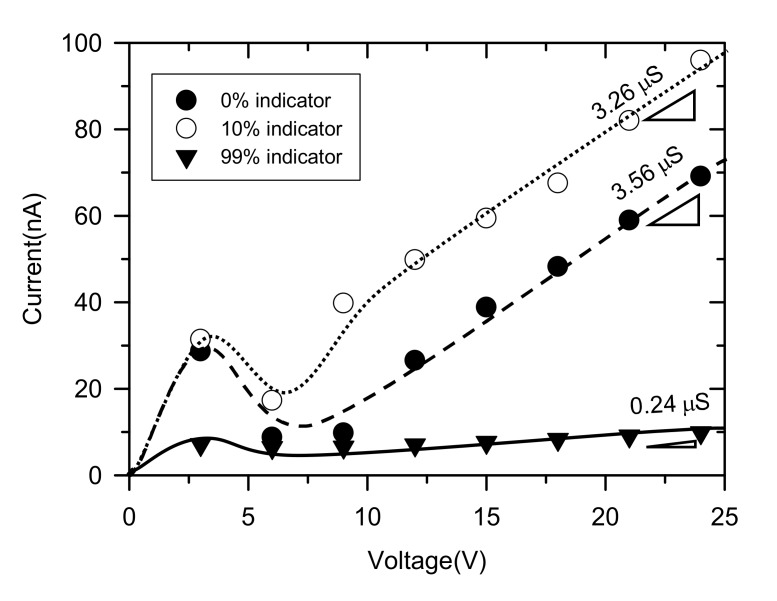
Current-voltage response in perm-selective ion transportation depending on the concentration of universal pH indicator. Overlimiting conductance (slope of linear region) significantly decreased at high concentration of indicator.

In order to check the concentration of universal pH indicator, *I*-*V* characteristics were measured. −*V* was applied to the main microchannel from 0 V to 25 V at 0.2 V/20 s, while the buffer microchannel was electrically grounded. External flow rate into both microchannels was 0.2 μL/min. As shown in Figure 2, the concentration of universal pH indicator resulted different *I*-*V* characteristics, especially overlimiting conductance values (i.e., the slope of overlimiting current regime). The conductance had a maximum value with 0 % indicator, meaning all of H^+^ contributed to the pH changes in the main microchannel. However, H^+^ from HCl and other cationic species from pH indicator simultaneously passed through the membrane as the concentration of pH indicator increased. As a result, one can obtain lower overlimiting conductance with 10% and 99% pH indicator concentration than 0% indicator as shown in Figure 2. The color changes were barely observed under 10% concentration so that we conducted further experiments with 10% of pH indicator mixture.

### 3.2. pH Gradient Generation by Proton Mass Transport

**Figure 3:**
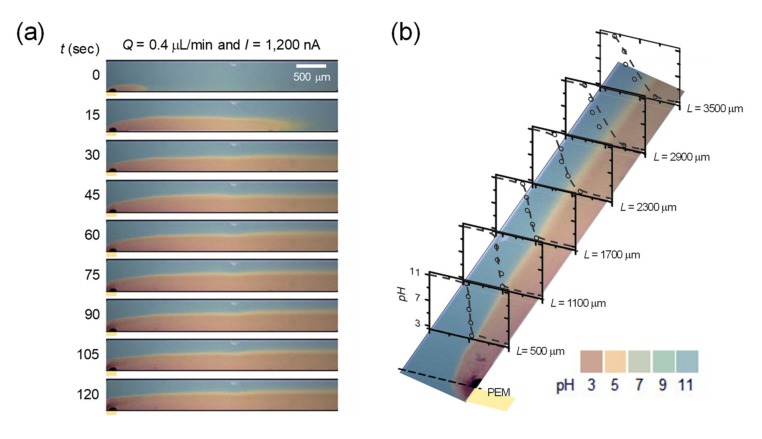
(**a**) Time-revolving snapshots of pH gradient generation along the microchannel. (**b**) The pH gradient as a function of distance from the nanojunction. The gradient flattened by the transversal diffusion of the H^+^ ion.

In the device shown in Figure 1a, 10 mM of NaOH (pH = 11) and 10 mM of HCl (pH = 3) were injected into the main and the buffer microchannel, respectively at the flow rate of 0.4 μL/min. In the meantime, external constant current of 1200 nA was applied through the proton-exchange membrane. 1200 nA was chosen just an example of good-looking presentation. Under these conditions, only H^+^ can be pumped into the main microchannel so that the pH gradient started to generate in longitudinal direction by diffusion. Thus, the color of solution in the main microchannel turned into reddish from the proton-exchange membrane. Since the Reynolds number in the main microchannel is around 0.1, one can expect a laminar flow regime in the channel as shown in Figure 3a. See the Supplementary Video S1. Furthermore, the sharp gradient near the membrane (see inset graph at *L* = 500 μm) gradually flatten along the transverse direction (see inset graph at *L* = 3500 μm) as shown in Figure 3b. Therefore, one can choose the shape of gradient at one’s discretion by placing a target object at desirable distances from the membrane.

**Figure 4:**
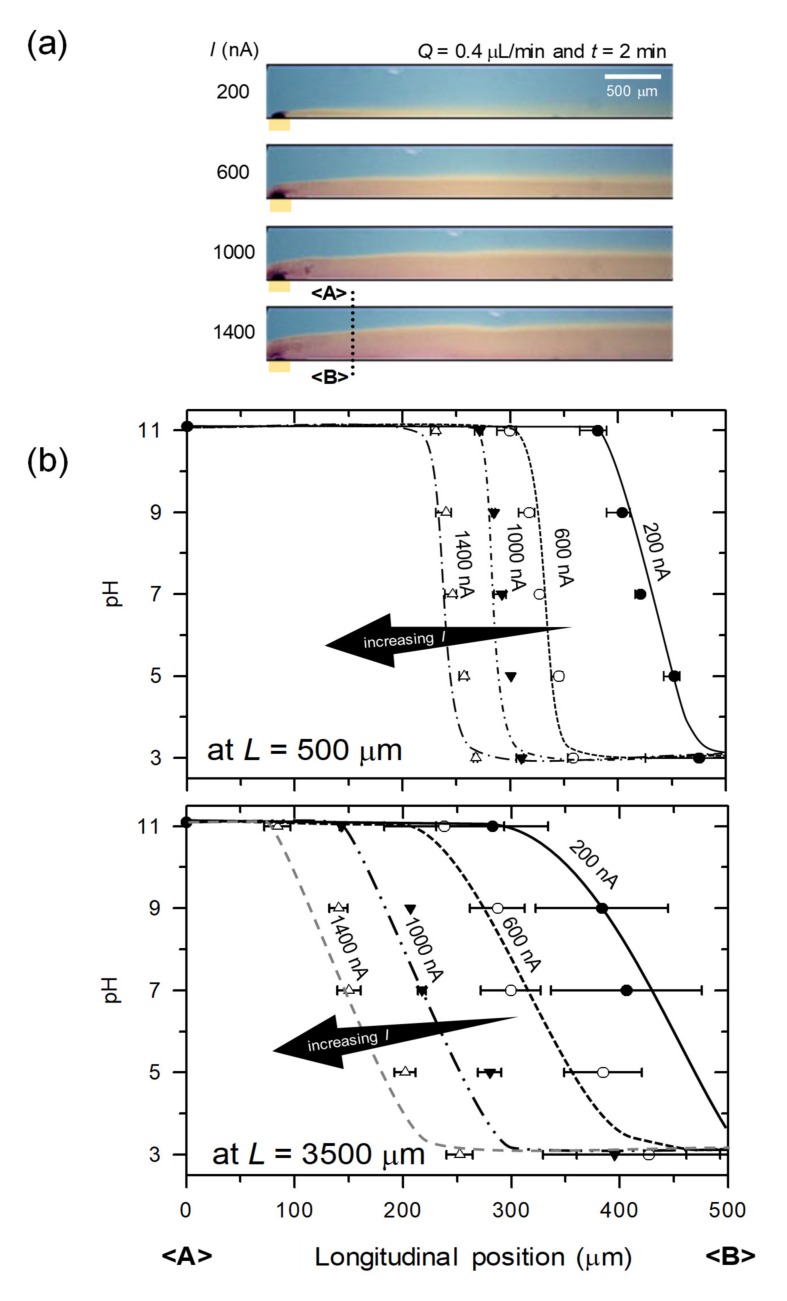

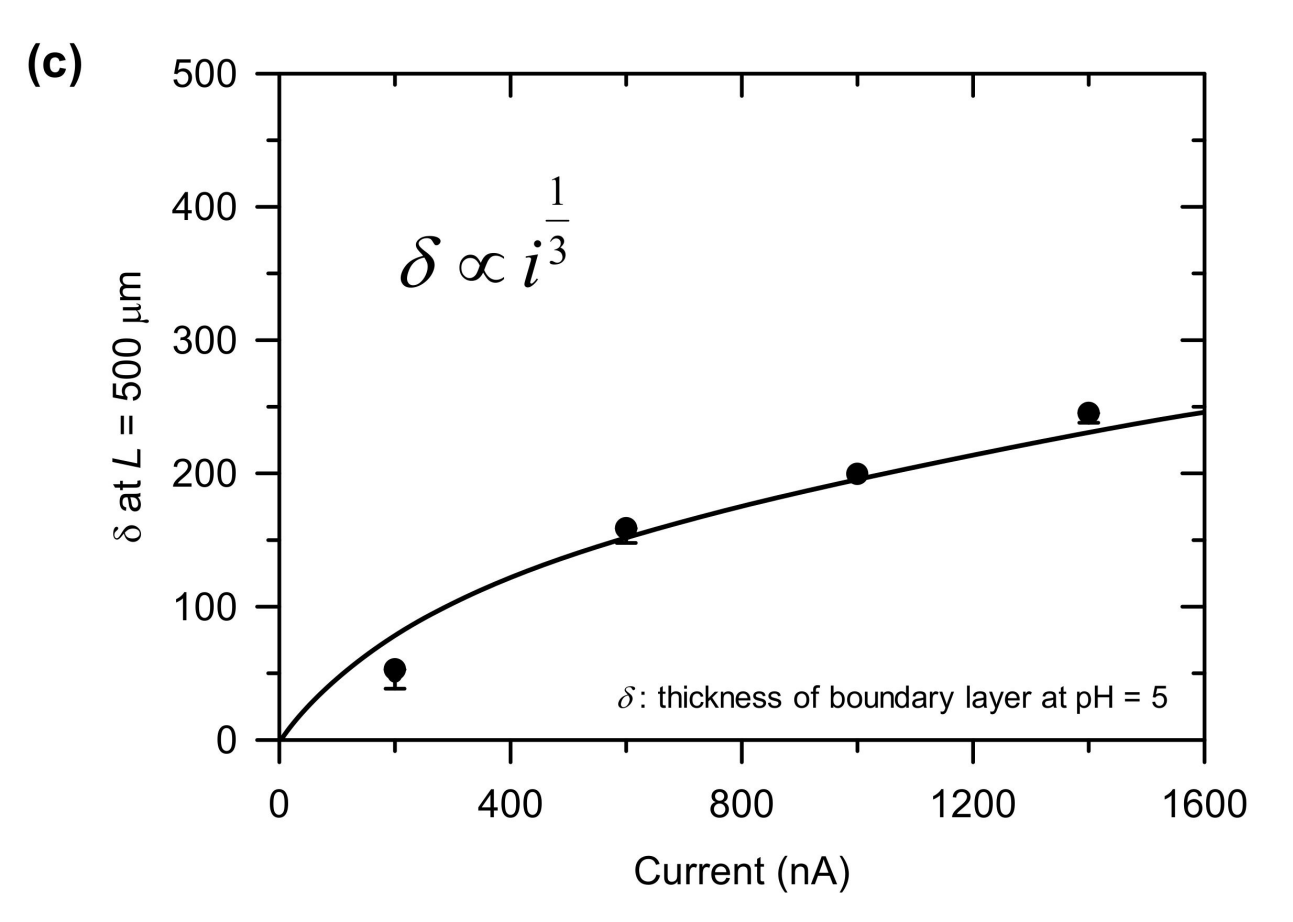
(**a**) The generation of pH gradient in the main microchannel as a function of the applying electric current. (**b**) Plots of pH gradient at *L* = 500 μm and *L* = 3500 μm as a function of applied current. Higher current resulted in steeper gradient. (**c**) Plot for the scaling law between current and the boundary layer thickness. The boundary layer was defined as the horizontal distance from the bottom of microchannel to the stream of pH = 5.

The first useful demonstration of pH gradients was a tunability by controlling the proton mass transport through the perm-selective membrane. The amount of proton mass transport into the main microchannel was set by adjusting the current value. The device produced various shape of pH map in the main microchannel at each current value as shown in Figure 4a. The images were captured at 2 min after applying each current. The fixed external input flow rate of 0.4 μL/min was applied from the left reservoir. At a glimpse, the thickness of reddish region (i.e., low pH regime) increased as the current increased. In order to quantify the pH values, image analysis by HSV model was performed. The interface between low and high pH shifted to center of the main microchannel as current increased (i.e., proton mass transportation increased) as shown in Figure 4b. The color code of five particular pH values (pH = 3, 5, 7, 9, and 11) were automatically tracked by customized MATLAB program. The *x*-axis is longitudinal position in the main microchannel from <A> to <B>. The sharp gradients dispersed as the gradient flew along the microchannel by comparing the measurement at *L* = 500 μm and 3500 μm. The pH values measured near the nanojunction (i.e., *L* = 500 μm) has smaller deviation than one measured far from the nanojunction (i.e., *L* = 3500 μm), which indicated more severe diffusion as a function of distance from the nanojunction. The correlation between applied current (*i*) and pH variation was analyzed from a convection-free diffusion equation, *dc_H_*/*dt* = *D_H_*∇^2^*c_H_* where *c_H_* and *D_H_* are the concentration and diffusivity of proton. Note that convection is negligible in longitudinal direction. By the definition of current, *i* = *dq*/*dt* = 2*eFdc_H_*/*dt* = 2*eFD_H_*∇^2^*c_H_*, one would obtain the linear correlation between *i* and *D_H_*. Since the boundary layer thickness, *δ* is proportional to 1/3th power of diffusivity [50], one finally got the relation as *δ* ∝ *ι*^1/3^. This relation confirmed in Figure 4c. Note that *δ* in Figure 4c is the thickness of boundary layer where pH = 5. In addition, the pH gradient dynamically expanded (or shrank) as the external current increased (or decreased), vice versa. See the Supplementary Video S2.

### 3.3. Tunable pH Gradient Generation by External Input Flow Rate

**Figure 5:**
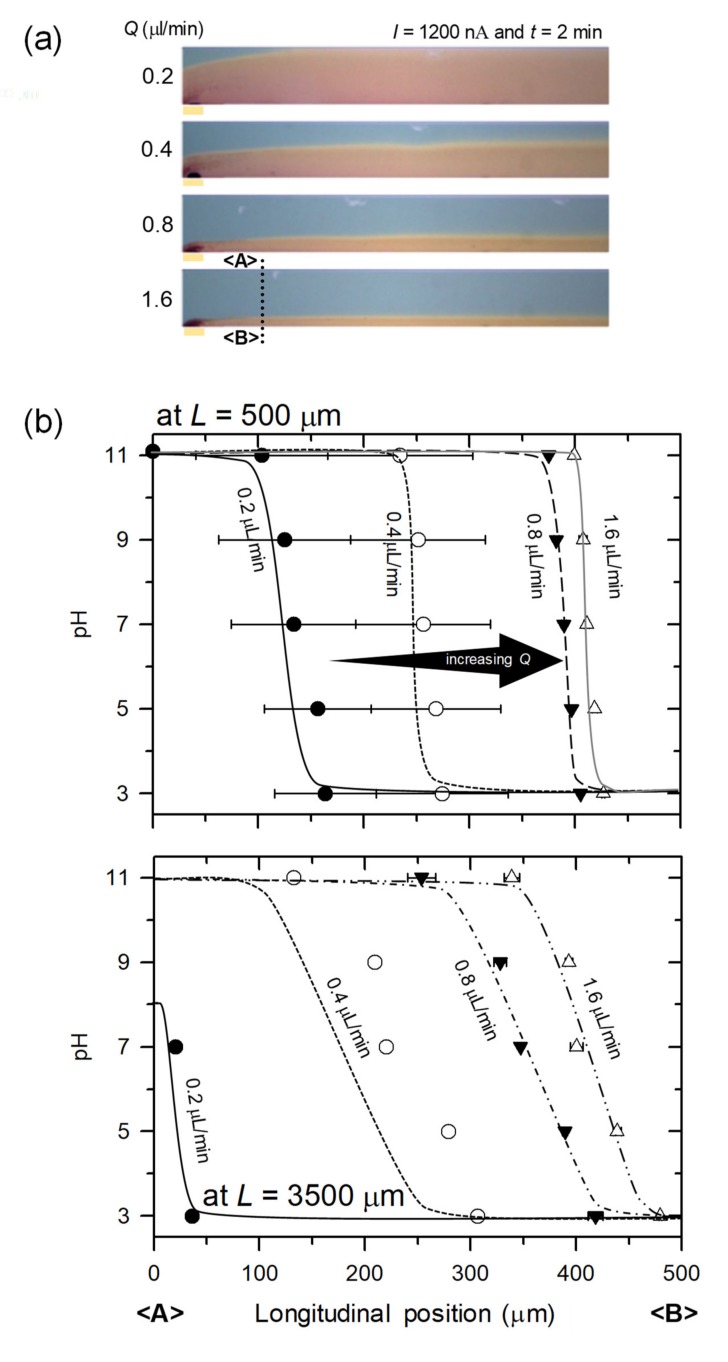

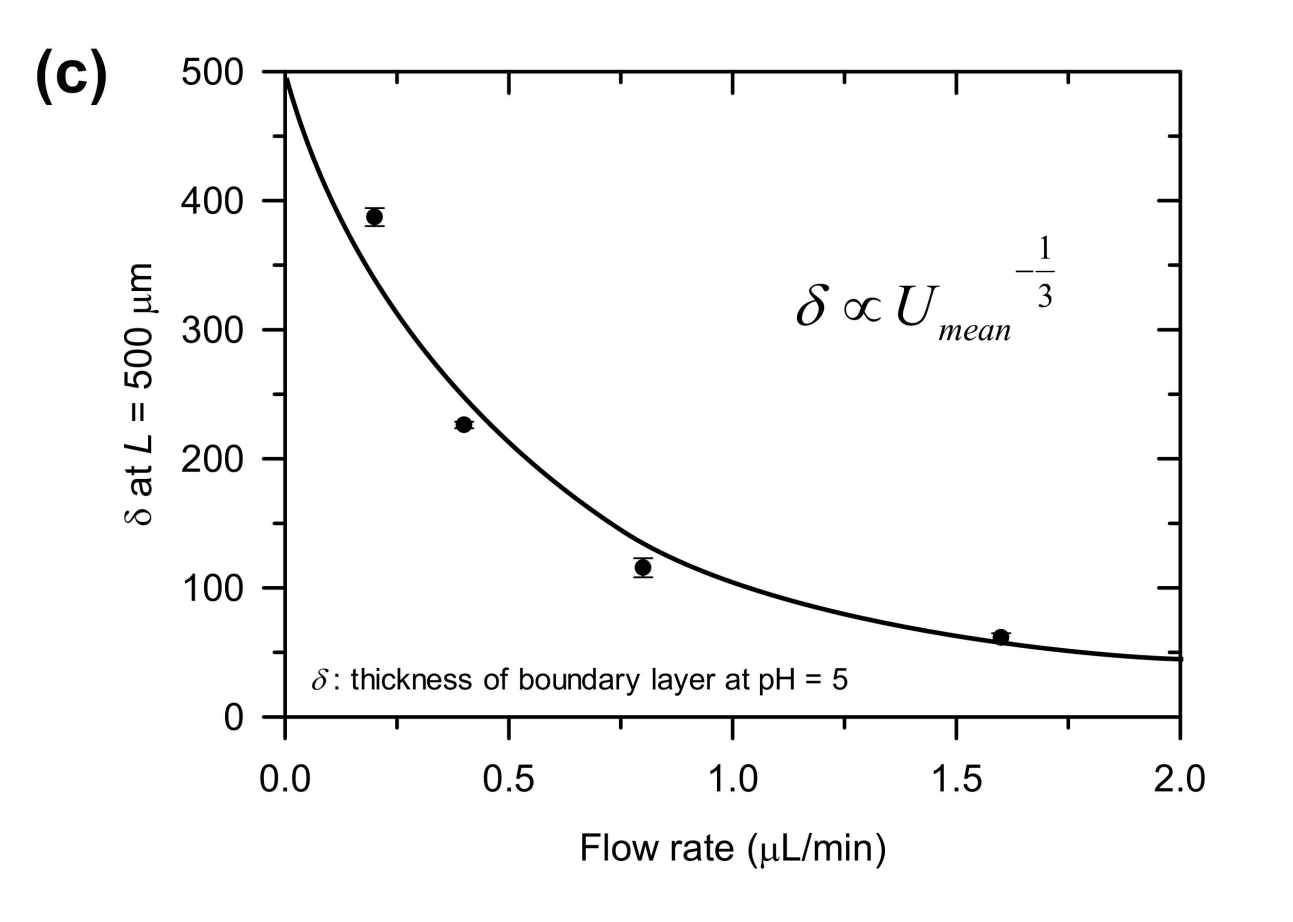
(**a**) The generation of pH gradient in the main microchannel as a function of the external flow rate. (**b**) Plots of pH gradient at *L* = 500 μm and *L* = 3500 μm as a function of flow rate. Higher flow rate resulted steeper gradient. (**c**) Plot for the scaling law between mean flow velocity and the boundary layer thickness. The boundary layer was defined as the horizontal distance from the bottom of microchannel to the stream of pH = 5.

The second demonstration of tunability of pH gradient was conducted by controlling the external input flow rate in both microchannels. The amount of flow rate into the main and buffer microchannel could change the pH variation. The various shapes of pH map in the microchannel at each flow rate were observed as shown in Figure 5a. The images were taken at 2 min after applying the external input flow rate and the current of 1200 nA. As mentioned above, HSV model was performed to analyze the pH values.

When the solution flew through the main microchannel with different external input flow rates, its pH profile redistributed as shown in Figure 5b. The interface between low and high pH shifted as flow rate varied. Figure 5c showed the relation between thickness of the boundary layer and flow rate. This could be explained by Sherwood number (*Sh*), $\frac{W^{2}U_{mean}}{L_{n}D_{eff}}$ where *W* is the half-width of the microchannel, *L_n_* is the length of the nanoporous medium, *D_eff_* is effective diffusivity of analytes and *U_mean_* is the average velocity of external flow [50]. Since the boundary layer thickness, *δ* can be expressed as $\frac{\delta }{W}\approx {\left(\frac{3}{Sh}\frac{y}{L_{n}}\right)}^{\frac{1}{3}}$ under these conditions, one finally got the relation as *δ* ∝*U_mean_*^−1/3^. This relation confirmed in Figure 5c. Note that *δ* in Figure 5c is the thickness of boundary layer where pH = 5.

### 3.4. Nanofluidic Sensing of pH Stimulation

**Figure 6:**
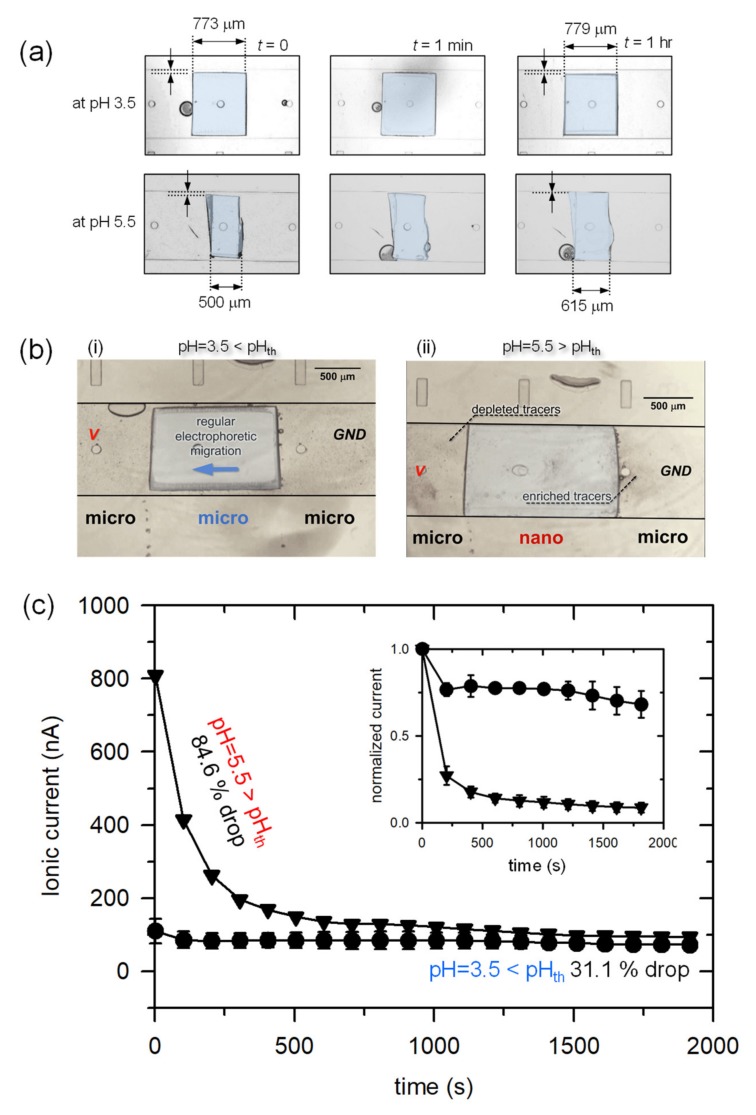
(**a**) The time-revolving snapshot of hydrogel swelling in different pH solutions (pH = 3.5 and 5.5). (**b**-**i**) The tracers moved as regular electrophoretic migration at pH = 3.5 which prevented the swelling. In this case, the hydrogel acted as a microjunction. (**b**-**ii**) The generation of strong vortices at pH = 5.5. In this case, the hydrogel played as a nanojunction so that the ion concentration polarization (ICP) can be formed. (**c**) *i*-*t* plot with constant applied voltage in both pH conditions and the inset showed the normalized current values. The current dramatically dropped only when the hydrogel completely swelled.

In previous section, we demonstrated the tunable pH generation only by the electric current (i.e., proton mass transport) via ICP phenomenon without pumping any chemicals. Inversely, one can sense surrounding pH alteration by examining whether ICP phenomenon occurs or not. To achieve this, pH-responsive hydrogel was patterned in the middle of microchannel as shown in Figure 1b. The hydrogel used in this work significantly swelled when it immersed a solution of pH over 4.5. Otherwise, it keeps its original volume. Therefore, the width of hydrogel was almost unchanged at pH = 3.5, while it expanded by 23% after 1 h in the solution of pH = 5.5 as shown in Figure 6a. When the swollen hydrogel blocked a microchannel, the configuration of microchannel was changed into micro-nano-micro-connection because the hydrogel possesses a number of nanopores inside [26,29,51]. Consequently, one can expect ICP phenomenon with the swollen hydrogel. This change was verified by both visualization and electrical measurement. First, the 1st kind of electroosmotic flow (EOF) was only observed at pH below 4.5. The tracers followed regular electrophoretic migration through micro-micro-micro-connection as shown in Figure 6b-(i). However, the “nano” junction appeared in Figure 6b-(ii) can initiate ICP. The signature of fluid motion in ICP layer is the formation of the ion depletion zone and strong vortices [4,9]. EOF generally refers the motion of liquid induced by an applied potential across a porous material, membrane and microchannel. Usual (or 1st kind of) EOF is governed by Smoluchowski relation which correlates the fluid velocity is linearly proportional to the applied electric field. However, EOF becomes 2^nd^ kind when the system has non-uniform properties of concentration, electric field and zeta potential, etc. Usually 2^nd^ kind of EOF has circulation (or vortical) flow. Because ICP creates non-uniform concentration field and electric field to lead a circulation flow, the appearance of circulation would be the key evidence of ICP phenomenon. At the anodic side of hydrogel, the tracers started to rotate and be repelled toward reservoir simultaneously. At the cathodic side of hydrogel, the tracers moved toward the hydrogel, which represented an enrichment zone. See the corresponding Supplementary Video S3 on Figure 6b.

Another fingerprint of ICP is *I*-*t* response. Due to the formation of ion depletion zone which has extremely high electrical resistance [52,53,54], the initial current should sharply drop as a function of time. These changes were measured as shown in Figure 6c. A constant voltage of 10 V was applied in both pH conditions. The current dropped immediately after the application of voltage in the case of pH = 5.5. Comparing to the initial value, it dropped 84.6% after 2000 s. Otherwise, it slightly dropped from its initial value (31.1% drop). The reason why it slightly dropped in case of pH = 3.5 was that ICP also formed when a micro- and a nano-junction coexist in parallel [55,56,57,58]. While one expects a higher initial current in a micro-micro-micro-connection than one in a micro-nano-micro-connection by considering cross-sectional area, it involves complex interactions such as Donnan concentration of nanoporous material [51,59], surface charge of microchannel [13], the concentration of electrolyte (i.e., Debye length) [2,55], and the transversal dimension of hydrogel itself, etc. Thus, normalized current values with respect to each initial current value were plotted in the inset of Figure 6c. If further developed (e.g., multiplexed design with hydrogels that react multiple pH values), one can utilize this scheme for a simple pH meter.

## 4. Conclusions

ICP phenomenon has been utilized for various interesting engineering applications using zero-Reynolds number characteristics such as portable scale desalination / purification devices, selective preconcentration devices of biomolecules. In those platforms, inevitable pH variation has been considered a significant nuisance that one should overcome. However, here we presented ways to utilize the pH changes instead of avoiding it. First, we demonstrated the effect of electric current on pH by controlling proton mass transportation. Proton-exchange membrane only transferred H^+^ into the main microchannel which was filled with basic solution so that the smooth pH gradient was generated by the combination of Laminar flow characteristics and the transversal diffusion of H^+^ ion. The pH gradient was generated without suffering from gas and contaminant generation due to water electrolysis. By adjusting electrical current (i.e., proton transportation) through the membrane, the gradient was shown to be tunable without complex addition of acidic or basic solutions. Secondly, we demonstrated that surrounding pH alterations were identified by examining the occurrence of ICP phenomenon. The alterations of surrounding pH can significantly change the electric current response using pH-responsive hydrogel which swells over pH_th_ = 4.5. The hydrogel patterned in the middle of a microchannel blocked and acted as a nanojunction when surrounding pH was greater than pH_th_. Then the current dramatically dropped because of the formation of an ion depletion zone. When pH < pH_th_, the hydrogel would allow the solution pass through the microchannel so that the current merely changed.

The strategies that presented in this work would be an basis for developing useful engineering applications such as a localized pH stimulation to biomolecules using tunable pH gradient generation and portable pH sensor with pH-sensitive hydrogel, *etc*.

## Supplementary Materials

The following are available online at https://www.mdpi.com/2072-666X/11/4/400/s1, Video S1: Experimental visualization of pH gradient generation, Video S2: Experimental visualization of tunable pH gradient generation, Video S3: The formation of ICP layer at pH > pH_th_.
